# Evaluating the Effect of Gender on Nursing Students' Attitudes Toward Violence Against Women by Metaphor Analysis: A Qualitative Study

**DOI:** 10.1111/phn.70006

**Published:** 2025-07-25

**Authors:** Emine Delimehmet, Emel Bahadır Yılmaz

**Affiliations:** ^1^ Department of Women's Studies Institute of Social Sciences Giresun Turkey; ^2^ Department of Women's Studies Giresun University, Institute of Social Sciences Giresun Turkey

**Keywords:** gender, metaphor, nursing students, violence against women, woman

## Abstract

**Objective:**

The objective of this study is to evaluate the effect of gender on nursing students' attitudes toward violence against women using metaphor analysis.

**Methods:**

The study was conducted with 64 nursing students from January to April of 2023. Metaphor analysis was used as a research method. In the Metaphor Identification Form on Violence Against Women, students were asked, “Violence against women is similar to …………………. Because…………………………….” and they were asked to complete the blank space in this sentence by explaining a metaphor and the reason for the metaphor. The study adhered to the COREQ checklist.

**Results:**

The four main themes that emerged from the male students' metaphors were as follows: (1) violence injures women, (2) the perpetrator of violence is not a human being, (3) violence threatens the future of society, and (4) violence must be resolved. Similarly, four principal themes were derived from the metaphorical expressions of female students: (1) violence is destructive and kills, (2) violence separates women from life, (3) the perpetrator of violence is weak and powerless, and (4) violence destroys society.

**Conclusions:**

Female students generated metaphors that were more solution‐oriented. Male students used negative metaphorical language to characterize the perpetrator of violence. It was proposed that the nursing curriculum be enhanced by incorporating relevant courses and implementing social responsibility projects.

## 1 Introduction

Nursing education should not only equip students with a comprehensive understanding of medical knowledge and skills, but also facilitate their ability to recognize and address social issues. The phenomenon of violence against women represents a significant social issue that also poses a considerable threat to the physical and psychological well‐being of individuals (Türkkan and Odacı 2024). This phenomenon should be taken into consideration during health service delivery as a situation that nurses may encounter. Nursing students' perceptions of this issue will influence their future professional practices and contribute to more informed decision‐making in patient care (Bahadır‐Yılmaz and Yüksel 2023). As a vital component of the healthcare sector, they bear a crucial responsibility for identifying this issue and implementing efficacious interventions.

Health workers lack sufficient knowledge about the actions they should take when witnessing violence or encountering a woman experiencing violence, highlighting the need for training (Arora et al. 2023). Furthermore, the study indicates that educational activities have a limited impact and that further research is required to gain a more comprehensive understanding of the subject matter (Kalra et al. 2021). In their study, Yücens and Kalkan Oğuzhanoğlu (2020) found that 61.7% of healthcare workers defined violence as solely physical and sexual, and 39.5% of them had experienced violence at least once in their professional lives. Some of the participants even stated that violence can be justified in certain situations. In the study conducted by Oztürk and Toprak (2017) with healthcare professionals, some participants indicated that they had not received training on violence against women and communication with victims of violence. The majority of the participants stated that they were either unaware of the Domestic Violence Screening Form or had never completed this form. Consequently, it is essential to ascertain the perceptions and attitudes of healthcare professionals with regard to violence against women, with the undergraduate education process serving as a pivotal point of reference.

A systematic review on the subject found that both nurses and nursing students exhibited a contemporary perspective on spousal violence against women, shifting away from traditionalist views and adopting a critical stance against such violence (Ağartıoğlu‐Kundakçı and Uysal‐Toraman 2023). The results of a study conducted with university students, including those enrolled in health department courses, indicated that the majority of students considered violence against women and domestic violence to be justifiable and could accept violence, albeit at a low level (Uğur et al. 2021). The education of nursing students in the area of violence against women, their desire to receive information on this subject, their exposure to violence, and their witnessing of violence have been identified as factors influencing their attitudes (Bahadır‐Yılmaz and Yüksel 2020). The findings indicated no significant correlation between grade level and students' knowledge or attitudes toward violence against women (Tokur‐Kesgin and Hançer‐Tok 2024). This indicates the necessity of incorporating undergraduate education on the subject. An awareness of this issue among students will facilitate the development of a more sensitive approach in their future practices, enabling them to adopt a perspective that encompasses gender equality and human rights (Bahadır‐Yılmaz 2018).

Learning effective intervention methods for victims of violence against women will enhance the quality of nursing students' communication with these individuals and improve their care processes. Identifying nursing students' perceptions of violence against women is important not only for individual health care provision but also for social change. Addressing this issue in the educational process will enable nurses to be more sensitive and effective in their future practice, with a positive impact on public health. Therefore, the inclusion of issues related to violence against women in the curriculum of nursing education is crucial for the development of a sense of social responsibility.

This study aims to evaluate the impact of gender on nursing students' attitudes toward violence against women using metaphor analysis. Research questions:

1. What metaphors do students use to describe violence against women?
2. What themes emerged from students' metaphors about violence against women?

## 2 Methods

In accordance with the Consolidated Criteria for Reporting Qualitative Research (COREQ) guidelines, the authors ensured a comprehensive description of the research processes at all stages of this study (Tong et al. 2007).

### 2.1 Research Design

This study was conducted between January and April 2023 and utilized metaphor analysis, a qualitative research method. To identify the metaphors, a semi‐structured interview form was used to assess the perceptions of 64 students regarding violence against women.

### 2.2 Research Team and Reflexivity

One researcher is enrolled in a Master's program in Women's Studies, while the other is an Associate Professor of Psychiatric Nursing in the Department of Nursing. The first researcher received training in qualitative research methodology. The second researcher holds a certificate in qualitative research and is currently pursuing postgraduate studies in this field.

### 2.3 Sample

In this study, the affinity sampling method, which is one of the purposive sampling methods, was employed. The affinity sampling method involves the use of a relatively small affinity sample for the purpose of conducting a detailed study of the subject matter (Baltacı 2018). Therefore, the population of the study consisted of 105 final‐year nursing students. The inclusion criteria were: (a) being a final‐year nursing student, (b) agreeing to participate in the study, and (c) being willing to engage in open communication. The exclusion criteria were: (a) incomplete completion of the questionnaire forms and (b) not being a native Turkish speaker. A total of 64 students were included in the study. The final sample consisted of 64 final‐year students who met the inclusion criteria. The literature suggests that the required sample size for qualitative studies ranges from six to eight participants in heterogeneous groups and from twelve to twenty participants in homogeneous groups (Bekele and Ago 2022).

### 2.4 Data Collection Tools

The data collection tools used in this study included a researcher‐designed socio‐demographic information form and a metaphor identification form on violence against women.

**The socio‐demographic information form** includes questions on age, gender, marital status, income status, family structure, and place of residence. It has been created by the researchers in accordance with the literature (Bahadır‐Yılmaz and Yüksel 2020; Tokur‐Kesgin and Hançer‐Tok 2024; Bahadır‐Yılmaz 2018).

**The metaphor identification form on violence against women** has been designed by the researchers to determine the students' metaphors on violence against women. In the form, students were asked “Violence against women is similar to …………………. Because…………………………….” and they were asked to complete the blank space in this sentence by explaining a metaphor and the reason for the metaphor. A metaphor explains a concept, phenomenon, or event by likening it to another concept, phenomenon, or event. This characteristic makes metaphor a powerful mental model, as it allows for the establishment of relationships between dissimilar phenomena and facilitates the projection of one mental scheme onto another (Saban 2008). Metaphor analysis is widely used for three distinct purposes in qualitative research. The first is to enhance the process, the second is to describe its development, and the third involves the use of metaphors to explain the outcome. In this study, it was employed to explain the process (Güneş and Fırat 2016).

### 2.5 Data Collection Process

Before implementation, the forms were evaluated by two experts, each holding a doctorate in their respective fields: psychiatric nursing and public health. This was done to guarantee the forms' validity and reliability. Afterwards, a pilot study was conducted with five students. After the evaluation process was completed, the forms were finalized.

Students received a detailed explanation of the research objectives in a face‐to‐face setting within the classroom during scheduled lessons. Subsequently, the forms were distributed along with an explanation of the required procedures for completion, including the identification of relevant metaphors. A brief definition of metaphor was provided. Students took approximately 15 min to complete the form.

### 2.6 Data Analysis

The collected data were analyzed in five stages, with different sources utilized to define these stages (Saban 2008; Baltacı 2017; Çelik et al. 2020).

#### 2.6.1 Reading Phase

Each student was assigned a number (Q1, Q2, Q3, etc.). The students' metaphors were repeatedly reviewed. By reading the metaphors and the reasons for the metaphors, it was aimed to understand what the students perceived about violence against women and what they tried to explain.

#### 2.6.2 Grouping of Metaphors

At this stage, small notes were added next to the students' explanations, and efforts were made to identify similarities. In forming these similarities, both metaphors and their explanations were considered. Subsequently, the metaphors were classified based on their shared characteristics.

#### 2.6.3 Main Theme Generation Stage

At this stage, the main themes were developed based on the shared characteristics of the grouped metaphors. The focus is on what message similar features convey. A review of the literature on the subject has been conducted. Ultimately, eight main themes were identified, with four emerging from the male students' responses and another four from the female students'.

#### 2.6.4 The Validation Phase

At this stage of the process, the Miles‐Huberman model has been employed. In order to guarantee the reliability of the dataset, it is essential to achieve consensus between the various coders. The similarity formula is used for consensus: “Δ = ∁ ÷ (∁ + 𝜕)×100.” In the formula, Δ: Reliability coefficient, ∁: The number of topics/terms on which there is consensus, ∂: Represents the number of topics/terms on which there is no consensus. According to the coding audit that gives internal consistency, it is expected that the consensus between the coders should be at least 80% (Baltacı 2017). According to the formula, the reliability has been calculated as “59 ÷ (59 + 5) × 100 = 92.2,” and the reliability has been found to be high.

#### 2.6.5 Presentation of Data

At this stage, the IBM SPSS Statistics 25 package program has been used to present the demographic characteristics of the students, such as age, marital status, income status, and so forth, and the metaphors written by the students as number, percentage, mean, and standard deviation. In order to give examples of expressions appropriate to the main and sub‐themes, students' expressions have been quoted. The codes of the students are shown in brackets to indicate to whom the quotations belong.

### 2.7 Rigor of the Study

It is accepted that the most appropriate criterion for assessing the rigor of qualitative studies is reliability. It suggests that the research should meet four criteria to ensure that the process is reliable. These are reliability, transferability, dependability, and confirmability. For reliability, long‐term participation was ensured, and member checks were conducted. For transferability, the reader was allowed to assess whether the topic was transferable to their own situation. For dependability, a comprehensive explanation of metaphor analysis was provided to facilitate another researcher to conduct a similar study. For confirmability, a systematic data analysis method was used and the strategies specified were adopted and adhered to this framework (Maher et al. 2018).

### 2.8 Ethical Dimension of the Research

The research was conducted in accordance with the ethical standards set forth by the Social Sciences, Science and Engineering Sciences Research Ethics Committee of a state university (approval date: 14/12/2022, approval number: 29/02). Institutional permission was obtained from the head of the nursing department where the study was conducted. The research was conducted in accordance with the ethical principles set forth in the Declaration of Helsinki. After the study was explained to the students, their written consent was obtained (Table 1).

**TABLE 1:** Combined criteria for reporting qualitative research (COREQ).

Item no.	Guide questions/description	Explanations
**Domain 1: Research team and reflexivity**		
**Personal characteristics**		
1. Interviewer/facilitator	Which author/s conducted the interview or focus group?	First author: Lecturer, Gendarmerie First Lieutenant Second researcher conducted the interview
2. Credentials	What were the researcher's credentials? e.g., PhD, MD	First author: MSc Second author: PhD
3. Occupation	What was their occupation at the time of the study?	First author: Lecturer, Crime Investigations Unit Second author: Associate Professor, Psychiatric Nursing
4. Gender	Was the researcher male or female?	Researchers are female
5. Experience and training	What experience or training did the researcher have?	First author has taken qualitative courses. Second author has taken qualitative courses, experience in qualitative research, and has qualitative studies
**Relationship with participants**		
6. Relationship established	Was a relationship established prior to study commencement?	Participants were students of second author
7. Participant's knowledge of the interviewer	What did the participants know about the researcher? e.g., personal goals, reasons for doing the research?	Students knew that second author was an associate professor in psychiatric nursing
8. Interviewer characteristics	What characteristics were reported about the interviewer/facilitator? e.g., Bias, assumptions, reasons, and interests in the research topic	Before data collection, students were informed about the purpose of the research
**Domain 2: study design**		
**Theoretical framework**		
9. Methodological orientation and Theory	What methodological orientation was stated to underpin the study? e.g., grounded theory, discourse analysis, ethnography, phenomenology, content analysis	This was a qualitative study
**Participant selection**		
10. Sampling	How were participants selected? e.g., purposive, convenience, consecutive, snowball	The affinity sampling method, one of the purposive sampling methods, was used
11. Method of approach	How were participants approached? e.g., face‐to‐face, telephone, mail, email	Face‐to‐face interviews were conducted with students
12. Sample size	How many participants were in the study?	Sixty‐four nursing students were included in the study
13. Non‐participation Setting	How many people refused to participate or dropped out? Reasons?	Forty‐one students did not volunteer to participate in the study
14. Setting of data collection	Where was the data collected? e.g., home, clinic, workplace	Data were collected in the classroom
15. Presence of nonparticipants	Was anyone else present besides the participants and researchers?	No
16. Description of sample	What are the important characteristics of the sample? e.g., demographic data, date	They were nursing students and volunteered to participate in the study
**Data collection**		No
17. Interview guide	Were questions, prompts, and guides provided by the authors? Was it pilot‐tested?	Detailed knowledge is given in the data collection section
18. Repeat interviews	Were repeat interviews carried out? If yes, how many?	No
19. Audio/visual recording	Did the research use audio or visual recording to collect the data?	Interviews forms were used. There is no audio or visual recording
20. Field notes	Were field notes made during and/or after the interview or focus group?	There is no field note
21. Duration	What was the duration of the interviews or focus group?	It took approximately 15 min for the students to fill out the form
22. Data saturation	Was data saturation discussed?	Yes, it was
23. Transcripts returned	Were transcripts returned to participants for comment and/or correction?	No
**Domain 3: analysis and findings**		
**Data analysis**		
24. Number of data coders	How many data coders coded the data?	Two researchers
25. Description of the coding tree	Did the authors provide a description of the coding tree?	Themes and metaphors have been presented in results and tables
26. Derivation of themes	Were themes identified in advance or derived from the data?	Themes have been identified in results
27. Software	What software, if applicable, was used to manage the data?	It has been defined in data analysis
28. Participant checking	Did participants provide feedback on the findings?	No control
**Reporting**		
29. Quotations presented	Were participant quotations presented to illustrate the themes/findings? Was each quotation identified? e.g., participant number	Yes, quotations have been presented in results
30. Data and findings are consistent	Was there consistency between the data presented and the findings?	Yes, there was
31. Clarity of major themes	Were major themes clearly presented in the findings?	Yes, they are
32. Clarity of minor themes	Is there a description of diverse cases or a discussion of minor themes?	Yes, there is

## 3 Results

### 3.1 Socio‐Demographic Findings

Among all students, 31 were female and 33 were male. The mean ages of female and male students participating in the study were 20.45 ± 1.48 and 22.58 ± 2.79, respectively, and there was a statistically significant difference between them (*p* < 0.05). All of the students are single. The analysis revealed no statistically significant difference between female and male students with regard to income status, family structure, and place of residence (*p* > 0.05, Table 2).

**TABLE 2:** Socio‐demographic findings.

Characteristics	Female (*n* = 31)		Male (*n* = 33)		Test value	*p* value
**Age (mean±SD)**	20.45 ± 1.48		22.58 ± 2.79		−3.763	0.000
	*n*	%	*n*	%		
**Marital status**						
Single	31	100.0	33	100.0		
Married	0	0.0	0	0.0		
**Income status**						
Income equals expense	19	61.3	17	51.5	3.437	0.179
Income is less than expenses	10	32.3	16	48.5		
Income is more than expenditure	2	6.5	0	0.0		
**Family structure**						
Nuclear	25	80.6	24	72.7	0.558	0.455
Extended	6	19.4	9	27.3		
**Place of residence**						
City	16	51.6	12	36.4	3.827	0.148
Town	11	35.5	10	30.3		
Village	4	12.9	11	33.3		

### 3.2 Male Students' Metaphors

The male students' metaphors were analyzed, and four main themes were identified. The four main themes that emerged from these metaphors are as follows: (1) violence injures women, (2) the perpetrator of violence is not a human being, (3) violence threatens the future of society, and (4) violence should be resolved (Table 3).

**TABLE 3:** Male students' metaphors of violence against women.

Themes	Metaphors	*n*	%
The perpetrator of violence is not a human being	A person of weak character (4), an entity that is not human (3), psychological disturbance (2), unscrupulousness (2), bigotry/ignorance (2), an act that is despicable (1), an action that is wrong (1), an expression of inner evil (1), bigotry that is devoid of humanity (1)	17	51.5
Violence threatens the future of society	Violence against all (1), cutting one's own breath (1), spring devoid of flowers (1), forest bereft of trees (1), tree pruning (1), violence against everyone (1), domestic violence (1), assaulting a mother (1), lack of life (1)	9	27.2
Violence injures women	Withering a flower (1), breaking a bird's wing (1), plucking a flower (1), flower left without water (1), hope left unfinished (1)	5	15.2
Violence must be resolved	An event that must end (1), capital punishment (1)	2	6.1
Total		33	

#### 3.2.1 Main Theme 1: Violence Injures Women

Within this theme, students used metaphors such as withering a flower, breaking a bird's wing, plucking a flower, and dehydrating a flower. These metaphors define that violence harms women.

Q3: “A bird cannot fly when its wing is broken, and a woman's wing is broken when she is subjected to violence.”

Q57: “A woman who is subjected to violence may continue to exist, but her quality of life is diminished, and she may appear to wither day by day, as though she were a flower deprived of water.”

#### 3.2.2 Main Theme 2: The Perpetrator of Violence Is Not a Human Being

Under this theme, students used metaphors such as a weak person, an inhuman being, unscrupulousness, meanness, and cowardice. With these metaphors, they tried to define the perpetrator of violence.

Q18: “I think men who commit violence against women are weak.”

Q58: “The inhuman person harms those who are vulnerable and under his/her authority.”

#### 3.2.3 Main Theme 3: Violence Threatens the Future of Society

The students used various metaphors, such as cutting one's own breath, a forest without trees, and a spring without flowers. They also asserted that violence has a widespread impact not only on women but also on the broader social fabric in which they live.

Q7: “Women are human and we are all human beings, all the evil done to them is also done to us.”

Q54: “Violence against women can be defined as the absence of another life.”

#### 3.2.4 Main Theme 4: Violence Must be Resolved

Of the male students, only two employed metaphorical language to illustrate their stance on the resolution of violence. They proposed that such violence should be regarded as a finite event, akin to capital punishment.

Q24: “Men and women have the same rights.”

Q64: “The argument is made that the absence of deterrent penalties results in a lack of compliance with the regulations, and therefore, the necessity for clear and precise provisions.”

### 3.3 Female Students' Metaphors

The analysis of metaphors used by female students revealed four main themes. These themes are as follows: (1) violence is destructive and kills, (2) violence separates women from life, (3) the perpetrator of violence is weak and powerless, and (4) violence destroys society (Table 4).

**TABLE 4:** Female students' metaphors on violence against women.

Themes	Metaphors	*n*	%
The perpetrator of violence is weak, powerless	weakness (3), an animal (2), dehumanization (1), weakness and powerlessness (1), incomplete puzzle (1), show of strength by powerless people (1), pepper (1), metamorphosis (1)	11	35.5
Violence separates women from life	Plucking a blossoming flower (2), plucking a rose from its branch (1), withering a flower (1), constantly plucking the scab of a wound (1), starfish (1), a tongue‐tied person (1), pressure and helplessness (1)	8	25.8
Violence is destructive and kills	Murder (2), fire (1), damage to the soul (1), crime (1), criminality (1), bomb (1)	7	22.6
Violence destroys society	Breaking the heart (1), indication of an undeveloped society (1), a generation (1), hatred of men (1), hatred and anger (1)	5	16.1
Total		31	

#### 3.3.1 Main Theme 1: Violence Is Destructive and Kills

Female students produced metaphors such as murder, fire, crime, cruelty, and bomb. With these metaphors, they revealed how destructive violence is.

Q6: “The life of a woman who has experienced violence in all its forms is devoid of meaning. The woman has become akin to a corpse, exhibiting no desire to continue living.”

Q19: “All forms of violence, whether directed at a woman or a human being, are manifestations of cruelty and inhumanity.”

#### 3.3.2 Main Theme 2: Violence Separates Women From Life

Female students employed metaphorical language, likening the act of violence against women to the act of plucking a blossoming flower, plucking a rose from its branch, or withering a flower. They asserted that violence has the effect of separating women from life.

Q10: “Just as a rose withers when it is torn from its branch, women are similarly afflicted by the effects of violence.”

Q40: “When individuals are unable to exert the level of pressure they desire on a woman, they experience a sense of rejection and resort to violence as a means of asserting their dominance. Once this point is reached, the woman is rendered helpless.”

#### 3.3.3 Main Theme 3: The Perpetrator of Violence Is Weak and Powerless

Female students highlighted that the perpetrators of violence are often perceived as helpless individuals, with metaphors such as helplessness, animal‐like qualities, and traits of meanness or ungratefulness.

Q13: “Individuals who do not see themselves as equal to their spouse are no different from animals.”

S50: “The individual who perpetrates violence assumes a distinct identity at the moment of violence, thereby losing their usual identity.”

#### 3.3.4 Main Theme 4: Violence Destroys Society

Female students used metaphors such as breaking the heart, indicators of an undeveloped society, and generation. With these metaphors, they expressed that violence destroys not only women but also society.

Q21: The heart is responsible for maintaining the body's upright position. A rupture of the heart precludes the possibility of survival. Similarly, the female plays a pivotal role in the sustenance of society. “If women are subjected to violence, society is unable to survive.”

Q44: “It is an indicator of lack of civilization.”

## 4 Discussion

In this study, the metaphors used by male and female nursing students to describe violence against women were analyzed. Four primary themes emerged from the male students' metaphors: The four main themes that emerged from the male students' metaphors were as follows: (1) violence injures women, (2) the perpetrator of violence is not human being, (3) violence threatens the future of society, and (4) violence must be resolved. Similarly, four principal themes were derived from the metaphorical expressions of female students: (1) violence is destructive and kills, (2) violence separates women from life, (3) the perpetrator of violence is weak and powerless, and (4) violence destroys society.

A review of the research findings suggests that both male and female students oppose violence against women, recognizing its detrimental impact on both women and society. A review of the literature reveals that gender is a factor influencing the knowledge and attitudes of nursing students with regard to violence against women. Specifically, male students are found to demonstrate lower levels of awareness and understanding than their female counterparts (Tokur‐Kesgin and Hançer‐Tok 2024; Pinar and Pınar 2022; Simsek and Ardahan 2020). A study conducted with nursing students in Türkiye found that male students were more likely to justify wife beating and believed that women who were beaten benefited from the beating (Tekkas‐Kerman and Ozturk 2022). Nevertheless, the findings of a recent study indicated that there was no discernible impact of students' gender on their attitudes toward violence against women (Yanıkkerem and Esmeray 2023). The current cohort of male nursing students evinces a heightened awareness of the potential for violence to be averted and of their own crucial role in the prevention of violence against women (Cerit and Alp‐Yılmaz 2023). It seems reasonable to posit that the inclusion of courses on this subject in nursing education, the undertaking of awareness‐raising activities, and the provision of gender equality courses may have contributed to this positive development.

A metaphor study conducted with university students to examine their perceptions of women found that female participants generated positive metaphors about themselves (e.g., bird of paradise, pearl, unique being), whereas male participants used metaphors with more negative connotations (e.g., devil, time bomb, monster, volcano, and rain) (Sargın 2018). In the study conducted by Günay and Odabaşı‐Koç (2021), female and male students employed positive metaphors to describe women, including terms such as “fragile,” “life source,” “life,” “flower,” “angel,” “love,” and “heart.” In contrast, male students utilized negative metaphors to define women, including references to “headache,” “devil,” “goat,” and “jackal.” The findings indicated that women employed more positive metaphors in defining themselves, whereas male participants utilized a greater number of negative metaphors. This suggests that the perception of violence against women may be influenced by gender‐based prejudices. In a study conducted to elucidate the perceptions of undergraduate students enrolled in the Social Studies Teacher Education program, it was observed that male students generated a greater number of negative metaphors (Sözer and Özkan 2014). In this context, the male nursing students in our study defined the man who inflicts violence on women with metaphors such as “non‐human being” and “helpless person,” which differs from the above studies. Similarly, in a study conducted with college students, students showed that they perceived abuse, especially physical abuse, committed by a man as more severe (Wilson and Smirles 2020). As a result of this, we can say that the female and male nursing students in this study were more sensitive to the issue and believed that violence against women was unjust.

It is unfortunate that gender inequality in society and traditional male roles have a negative impact on the perception of women and serve to legitimize violence against women (Altıntop and Adana 2019; Gürpınar et al. 2020). In fact, this mindset, that is traditional gender roles, causes women to legitimize violence (Ozalp and Albayrak 2022). Traditional gender roles also affect nursing students' attitudes toward violence against women (Tuncer and Birlikçioğlu 2024). It was observed that students who had attitudes that increased and tolerated violent behaviors against women had more traditional attitudes toward gender roles (Evcili and Daglar 2021). In this study, while none of the female students expressed support for this view, only one student stated that violence against women should only be employed when absolutely necessary. The objective of gender equality courses is to challenge traditional gender roles and prevent violence against women. Furthermore, the course is available as an elective within our faculty, with only approximately 50% of students pursuing this option. It is important to organize the curriculum so that all students take this course. Because in a study conducted with midwifery students at the same faculty, the gender equality course reduced the students' negative attitudes toward violence against women (Bahadır‐Yılmaz 2018). In another study, it was determined that education about women's and children's rights positively affected nursing students' attitudes toward gender roles (Turan 2022). In this regard, we can say that it is important to include courses on gender roles and violence against women in the education curriculum.

The findings of our study indicated that female participants placed greater emphasis on the destructive effects of violence compared to their male counterparts. Although male students primarily used metaphorical language to describe violent men, female students focused more on the destructive nature of violence and its broader societal ramifications. While both genders hold a negative view of violence, there are nevertheless discernible gender‐based differences. One of the participants in the study conducted by Gedik and Uzuner‐Akçıl (2020) asserted that women are the foundation of society and that society would cease to exist without women. This assertion is consistent with the metaphors that emerged in our study. In light of Çaldak's research (Çaldak 2021), which employed metaphor analysis to examine how individuals comprehend violence against women, identified the most prevalent metaphors among participants as “being an uncivilized behavior,” “weakness,” “monstrosity,” and “harming society.” The metaphors obtained in our study, such as the theme “The perpetrator of violence is not a human being” and the metaphors “an indicator of an undeveloped society,” have great similarities with the studies.

### 4.1 Limitations

This study has some limitations. The results of the study cannot be generalized to all Turkish students. Because the study was conducted in a single center. In addition, since the study was conducted in a small sample, it is suggested that similar studies be conducted in larger samples. Gender roles and violence against women also have a sociopolitical and cultural context. Since the subject can vary from culture to culture, it is also important to conduct comparative studies.

## 5 Implications for Nursing Policy

Despite the persistence of violence against women as a significant public health concern, its integration into university‐level nursing curricula in Turkey remains notably absent. It is essential for students to receive instruction through courses that enhance their understanding of violence against women, equip them with the necessary psychosocial skills to support victims, and facilitate the development of requisite competencies in case management. In addition, nursing students must know their roles and legal responsibilities before graduation. It is crucial to eradicate discrepancies in knowledge, awareness, and competencies based on gender inequality and to guarantee that male nurses assume proactive roles in clinical settings. It is imperative that all students are provided with the opportunity to engage with courses that address critical issues such as gender equality and violence against women. Such courses help challenge existing misconceptions and provide a comprehensive understanding of the multifaceted factors contributing to the perpetuation of violence. Furthermore, they facilitate the examination of social structures that shape attitudes and behaviors related to violence while promoting the teaching of egalitarian roles. These measures, when implemented on a long‐term basis, can effectively contribute to the resolution of the problem.

## 6 Conclusion

This study utilized metaphor analysis to assess the perceptions of male and female nursing students regarding violence against women. The findings indicate that both groups perceive violence against women as a significant issue. Female students were the only ones who generated more solution‐oriented metaphors and placed greater emphasis on the destructive effects of violence. Male students, on the other hand, used negative metaphorical language to characterize the perpetrator of violence. It has been determined that additional training is required on the effects of violence and potential solutions. It was proposed that the nursing curriculum should be augmented with the incorporation of pertinent courses and that social responsibility projects should be undertaken.

## Ethics Statement

Ethical approval of the research was received from Giresun University Social Sciences, Science and Engineering Sciences Research Ethics Committee. Informed consent was obtained from all participants who agreed to participate in the study. All stages of the study were conducted in accordance with the Declaration of Helsinki.

## Consent

Informed consent was obtained from all participants.

## Conflicts of Interest

The authors declare no conflicts of interest.

## Data Availability

The data that support the findings of this study are available from the corresponding author upon reasonable request.
